# Optical Metabolic Imaging of Mitochondrial Dysfunction on HADH Mutant Newborn Rat Hearts

**DOI:** 10.1109/JTEHM.2021.3104966

**Published:** 2021-08-16

**Authors:** Farnaz H. Foomani, Jason A. Jarzembowski, Soudeh Mostaghimi, Shima Mehrvar, Suresh N. Kumar, Mahsa Ranji

**Affiliations:** 1 Biophotonics LaboratoryDepartment of Electrical EngineeringUniversity of Wisconsin–Milwaukee 14751 Milwaukee WI 53201 USA; 2 Department of Pathology and Laboratory MedicineMedical College of Wisconsin 5506 Milwaukee WI 53226 USA; 3 Biophotonics LaboratoryDepartment of Electrical Engineering and Computer Science (EECS)ISENSE Institute, Florida Atlantic University 1782 Boca Raton FL 33431 USA

**Keywords:** Mitochondrial dysfunction, optical imaging, redox state, HADH, newborn rats

## Abstract

Background: Mitochondrial $\beta $-oxidation of fatty acids is the primary energy source for the heart and carried out by Hydroxy Acyl-CoA Dehydrogenase (HADH) encoded trifunctional protein. Mutations in the genes encoding mitochondrial proteins result in functionally defective protein complexes that contribute to energy deficiencies, excessive reactive oxygen species (ROS) production, and accumulation of damaged mitochondria. We hypothesize that a dramatic alternation in redox state and associated mitochondrial dysfunction is the underlying cause of Fatty Acid Oxidation (FAO) deficiency mutant, resulting in heart failure. Mitochondrial co-enzymes, NADH and FAD, are autofluorescent metabolic indices of cells when imaged, yield a quantitative assessment of the cells’ redox status and, in turn, that of the tissue and organ. Method: We utilized an optical cryo-imager to quantitively evaluate the three-dimensional distribution of mitochondrial redox state in newborn rats’ hearts and kidneys. Redox ratio (RR) assessment shows that mitochondrial dysfunction is extreme and could contribute to severe heart problems and eventual heart failure in the mutants. Results: Three-dimensional redox ratio (NADH/FAD) rendering, and the volumetric mean value calculations confirmed significantly decreased cardiac RR in mutants by 31.90% and 12.32%, in renal mitochondrial RR compared to wild-type control. Further, histological assessment of newborn heart myocardial tissue indicated no significant difference in myocardial tissue architecture in both control and severe (HADHA^e4−/−^) conditions. Conclusion: These results demonstrate that optical imaging can accurately estimate the redox state changes in newborn rat organs. It is also apparent that the FAO mutant’s heart tissue with a low redox ratio is probably more vulnerable to cumulative damages than kidneys and fails prematurely, contributing to sudden death.

## I. Introduction

Mitochondrial $\beta $-oxidation of fatty acids is the primary energy source for the heart and is carried out by Hydroxy Acyl-CoA Dehydrogenase (HADH) encoded trifunctional protein [1]. It is a hetero-octamer enzyme complex made up of 4 alpha and 4 beta subunits [2]. Mutation in HADH has devastating consequences, including sudden cardiac arrest and death in neonates [3], [4]. FAO defects, including trifunctional protein deficiencies, present complex and overlapping clinical symptoms that make diagnosis difficult. Though trifunctional protein deficiencies closely resemble long-chain 3-hydroxy acyl Co-A dehydrogenase deficiencies, it is far more severe with early and sudden death in neonates [5]. Long-chain acyl-CoA has also been shown to inhibit oxidative phosphorylation and increase the reactive oxygen species due to the lack of NADH [6], [7]. Further, fibroblasts from LCHADD patients show increased ROS levels in Long-chain Hydroxy Acyl-CoA Dehydrogenase patients due to significantly altered mitochondria [8].

Heart and kidneys are highly dependent on mitochondrial oxidative phosphorylation due to their high oxygen consumption [9]. Aerobic respiration, which involves the consumption of oxygen and ATP production, starts with pyruvate production from glucose through glycolysis [10], [11]. Pyruvate gets converted to acety1-CoA to supply the tricarboxylic acid (TCA) cycle to produce NADH, FADH_2_, and CO_2_. Electrons from NADH and FADH_2_ travel through complex I and complex II to complex IV of the electron transport chain (ETC), where they accept oxygen. Eventually, protons flow through complex V to trigger the conversion of ADP to ATP [12]. Cardiac muscles are dependent predominantly on aerobic respiration to supply their bioenergetic demand, while the mechanism by which ATP is produced in kidneys can be both aerobic and anaerobic. The respiration mechanism is dependent on the cell type in the kidneys. The anaerobic glycolytic pathway operates in cells with lower bioenergetic demand, such as glomerular cells, which depend significantly on glycolysis to produce ATP. However, the proximal tubules rely on oxidative phosphorylation as an ATP source [9], [11].

This manuscript presents the extent of mitochondrial damage in the trifunctional protein deficiency rat model using a quantitative optical cryoimaging technique. Optical fluorescence imaging as a diagnostic tool with high sensitivity and specificity enables us to discriminate between diseased and non-diseased tissue [7], [13], [14]. The development and utilization of optical imaging technologies such as fluorescence microscopy [15], confocal microscopy [16], and multiphoton microscopy [17] is a developing field in functional imaging of mitochondria and to assess various biomarkers related to their energy production level. To assess mitochondria function, quantification of two-electron carriers, NADH and FADH_2_, could provide information about the electron transport chain’s (ETC) substrate supply rate [18]. We used a custom-designed 3D fluorescence cryo-imager to monitor NADH and FAD (the oxidized form of FADH_2_) signals in mitochondrial [19]–[21]. NADH and FAD are autofluorescent and are measured without any exogenous labels by optical imaging techniques [22]. NADH/FAD’s ratio, also known as the redox ratio (RR), is associated with mitochondrial metabolic state and functionality [23], [24]. Any significant imbalance in the NADH and FAD concentrations would lead to cellular dysfunction and diseases [21]. Snap freezing organs in an isopentane bath cooled liquid nitrogen (−160°C) preserves the tissue’s metabolic state at the time of surgery.

Furthermore, cryo-temperature (−40°C) fluorescence imaging conserves the tissue’s metabolic state during imaging and provides a higher quantum yield of NADH and FAD than the room temperature measurements [13]. Importantly, cryo-imager provides 3D spatial distribution of NADH, FAD fluorescence intensities, and redox ratio of tissue [14], [19]. Our report suggests that the volumetric NADH/FAD redox ratio is significantly decreased (more oxidized) in the newborn heart carrying mutant trifunctional protein gene while the same animals’ kidneys showed only marginal difference compared to the littermate wild-type. Since NADH feeds electrons into complex I of the electron transport chain, while FAD does the same at complex II, the significant reduction in redox ratio seen in mutant hearts indicates extensive mitochondrial dysfunction that potentially contributes to heart failure.

## II. Material and Methods

### A. Animal Models

Wild-type Sprague Dawley rats were purchased from Charles River, Wilmington, MA, housed, maintained, and cared for according to the federal, state, and local guidelines of Animal Welfare ACT and Health research Extension ACT. All our experimental protocols were approved by the Institutional Animal Care and Use Committee (IACUC) of Medical College of Wisconsin (AUA5533). Transgenic HADHA^e4−/−^ mutant animals were generated using the CRISPR/CAS9 genome editing method (exon 4 excision – e4-/-) [25] done by the Transgenic Rat Core at Medical College of Wisconsin and backcrossed for three generations before experiments are conducted. Breeding pairs were housed in pairs during pregnancy and after delivery and were fed with purified rodent chow and water ad libitum. The genetic makeup of HADHA^e4+/+^ (wild-type control) and severe HADHA^e4−/−^ were confirmed by Polymerase Chain Reaction assay and DNA sequencing [manuscript under review]. The heart sample size of N = 6 for wild-type control, and N = 11 for severe HADHA^e4−/−^ were collected. For kidney samples, the number of the wild-type control and severe HADHA^e4−/−^ samples is N = 5 and N = 12, respectively.

### B. Experimental Protocol

#### 1) Tissue Isolation

Newborn pups (P0) between 3–6hrs were decapitated by guillotine method, and the heart and kidneys were surgically removed, placed in a cryotube, and quickly immersed into an isopentane bath cooled in liquid nitrogen. All pups were euthanized to collect organs within 30min time period, and the tissue samples were stored at −80°C freezer until processed further. Tissue samples were deidentified by the tissue preparer until all the analysis was completed. The tissue samples for this study came from littermates of 6 different breeding pairs, and all originated eight generations before from a single founder animal. For histological evaluations of heart and kidney tissue, FFPE (formalin-fixed paraffin-embedded) blocks were prepared at the Children’s Research Institute Histology core.

#### 2) Hematoxylin and Eosin Staining

Tissues from newborn pups were collected within 2hrs of birth. The newborn pups were decapitated, slit open the thoracic /abdominal cavity using sharp scissors. Heart and kidney were harvested rapidly and immersed in 4% neutral buffered formalin for 24hrs. Following dehydration, paraffin embedding, a 4-$5\mu \text{m}$ section was deparaffinized, rehydrated, and stained with Hematoxylin and eosin (H&E) staining [26]–[28]. Mason trichrome staining of heart tissue was done to estimate the extent of tissue infarction [29].

#### 3) Mitochondrial Density

Estimation of mitochondrial density was performed by standard immunohistochemistry method using an anti-Tomm20 antibody (Sigma-Aldrich, USA) on 4-$5\mu \text{m}$ formalin-fixed paraffin-embedded tissue sections. Mitochondrial density quantitation was done using Image J (NIH v1.51) on three different sections cut $50\mu \text{m}$ apart [30].

#### 4) 3D Optical Cryo-Imaging

**FIGURE 1.**
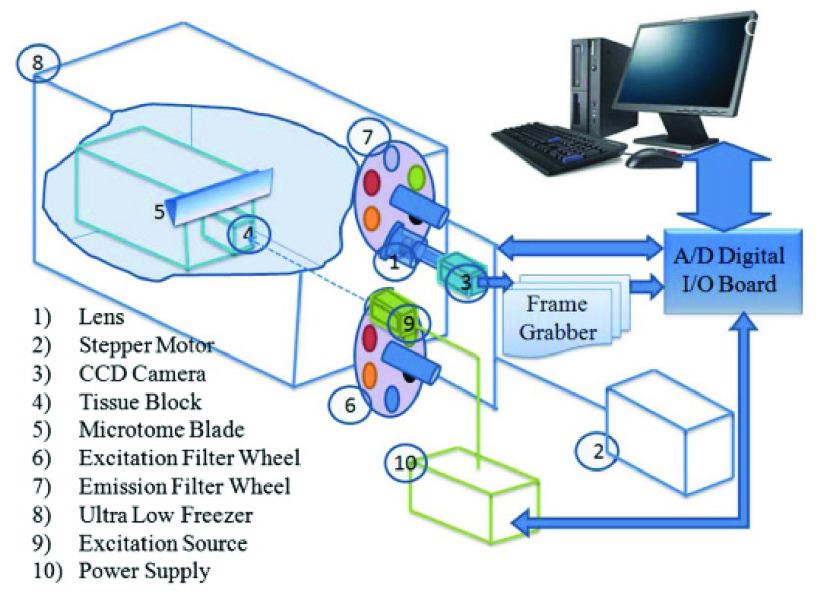
Schematic of Cryo-imaging system. This device sequentially slices the tissue, imaging the surface between each successive slice through up to five different channels. The images are then displayed and saved to a computer, where they can be processed to create 3-D renderings of the tissue [22].

The 3D cryo-imager is a custom-designed instrument designed and developed at the Biophotonics Lab, University of Wisconsin Milwaukee. A schematic of the Cryo-imaging system is shown in Figure 1. The snap-frozen tissues were stored at −80°C until the day of study. Afterward, these samples are embedded inside a freezer and then imaged at the cryogenic temperatures (−40°C) [16]. Imaging in lower temperatures guarantees a higher quantum yield of fluorescence imaging and preserves the tissue’s metabolic state at the time of freezing [7], [31]. The freezing and embedding protocol are described in detail in our previous studies [18]. The tissue was excited by a mercury arc lamp (200W lamp, Oriel, Irvine, CA), the excitation light passes through the excitation filters (350nm ±40nm for NADH; UV pass Blacklite, HD Dichroic, Los Angeles, CA, and 437±10nm for FAD; 440QV21, Omega Optical, Brattleboro, VT). A microtome located inside the freezer sequentially slices the tissue at $25\mu \text{m}$ thickness. The emitted autofluorescence signal is filtered before being recorded by a CCD camera (QImaging, Retiga R6, 16bit). Emission filters for NADH and FAD were 460nm±25nm, D460/50M Chroma, Bellows Falls, VT, and 537nm±25nm, QMAX EM510-560, Omega Optical, Brattleboro, VT, respectively. Image acquisition and computer control of the microtome motor and filter wheels are automated and controlled through LabVIEW software (2018 National Instruments).

**FIGURE 2.**
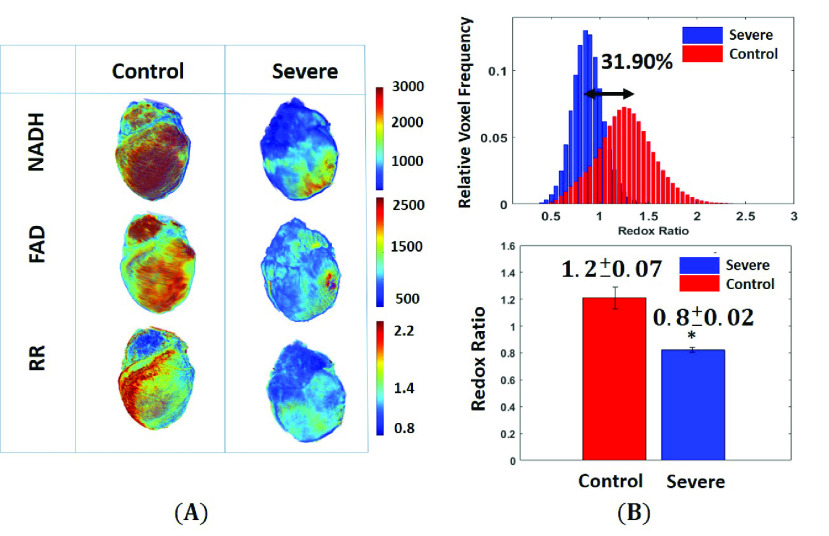
(A) Representative 3D reconstructions images of the mitochondrial NADH, FAD, and redox ratio (RR = NADH/FAD) from each of the two groups: Control and Severe (HADHA^e4−/−^). (B) Corresponding histogram distribution of the redox ratio from the two hearts is shown in panel (A). The percentages represent the difference between the mean value of the redox ratio histograms from all the samples. Hearts with severe (HADHA^e4−/−^) condition showed a 31.90% decrease in their mitochondrial redox ratio than the control group. The bar graphs represent the average values and standard deviation of the means of the histogram redox ratios for the two groups. Statistical analysis shows a significant difference between control vs. severe (HADHA^e4−/−^) (*p = < 0.001, t-test).

#### 5) Image Processing

NADH and FAD autofluorescence images (on average 150 slices per sample) from each group of kidneys and hearts were processed using MATLAB (The MathWorks, Inc., Natick, MA). A segmentation algorithm based on thresholding was applied to each slice to segment the region of interest on the tissue [17]. 3D images are obtained by stacking the slices in the z-direction. The ratio of NADH and FAD, the redox ratio, was calculated by dividing the NADH over FAD images voxel by voxel. The redox ratio histogram distribution of each kidney and heart sample was calculated, and the average of these histograms was measured for further statistical analysis according to equation (1).

\begin{align*} Mean~of~RR \!=\! \frac {1}{N_{x} \times N_{y}\times N_{z}}\! \sum \limits _{i=1}^{N_{x}}\sum \limits _{j=1}^{N_{y}} \sum \limits _{k=1}^{N_{z}} RR ~Volume(i,j,k)\!\! \\ {}\tag{1}\end{align*}

where $\text{N}_{\mathrm {x}}$, $\text{N}_{\mathrm {y}}$, and $\text{N}_{\mathrm {z}}$ are the number of pixels in the x, y, and z directions and RR is the redox ratio.

### C. Statistical Analysis

Statistical analyses were performed on the averaged redox ratio values as described previously [20], [21]. The assumption that the samples were drawn from a normally distributed population was checked by Kolmogorov-Smirnov test and confirmed. Tukey’s t-test analysis was performed using MATLAB to assess the quantitative differences between groups. The significant level was considered at P < 0.05.

## III. Results

Figure 1A represents the 3D rendering of NADH, FAD, and their ratio (RR = NADH/FAD) from representative hearts of each of the two groups (Control and Severe (HADHA^e4−/−^). Hearts with severe conditions (HADHA^e4−/−^) had a decreased RR compared to the control group reflecting a maximally oxidized metabolic status. Figure 1B shows the corresponding relative voxel frequency distribution of RR for the two representative samples in Figure 1A. The bar graphs represent the mean value of the histograms. The RR values confirmed an increased oxidized metabolic state in hearts with the severe (HADHA^e4−/−^) conditions group than the wild-type control. The severe group had a 31.90% decrease in RR compared to the control, a significant difference between the two groups (p < 0.001, with 95% confidence intervals [0.2497, 0.5214], Tukey’s t-test).

**FIGURE 3.**
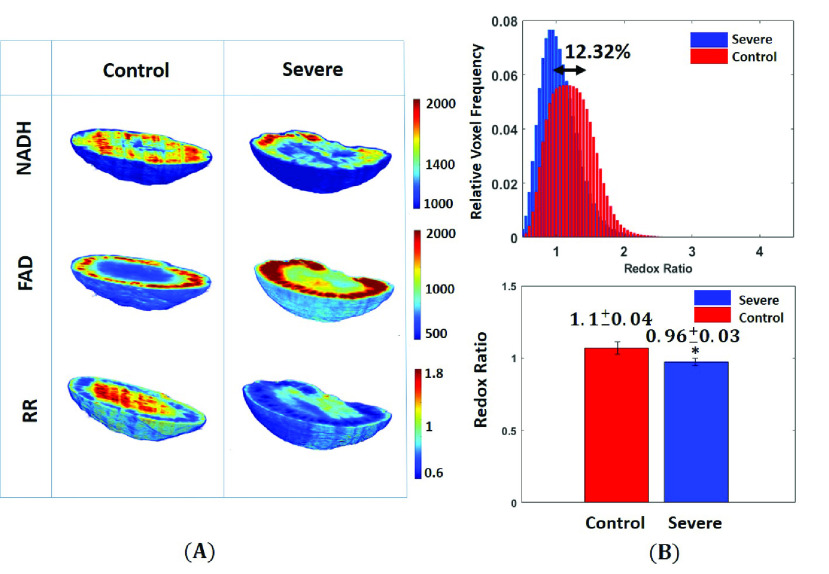
(A) NADH, FAD, and redox ratio 3D Cryo-images of representative kidneys from control and severe (HADHA^e4−/−^) groups. (B) Their corresponding redox ratio (NADH/FAD) histograms. The kidneys’ redox ratio in the severe (HADHA^e4−/−^) group dropped by 12.32% compared to the kidneys in the control groups. The bar plots represent mean and standard deviation values of the average redox ratio in histograms. * shows a significant difference in the severe (HADHA^e4−/−^) group when compared to the control group (p = 0.0127, t-test).

Representative cryoimages of kidney the NADH, FAD, and RR obtained from the control and severe (HADHA^e4−/−^) rat pups are shown in Figure 3A. The corresponding redox ratio histograms of the same three samples are illustrated in Figure 3B. The bar plots show the mean ± standard deviation of the RR histograms’ average value for the two groups. Comparing rat pups with severe (HADHA^e4−/−^) mitochondrial dysfunctionality to control, showed a lower NADH, higher FAD in the former group. Kidney tissue showed smaller RR (12.32% less with p = 0.0127, with 95% confidence intervals [0.0335, 0.2380]) than the heart tissue, suggesting a lower level of mitochondrial dysfunction in severe (HADHA^e4−/−^) conditions pups.

**FIGURE 4.**
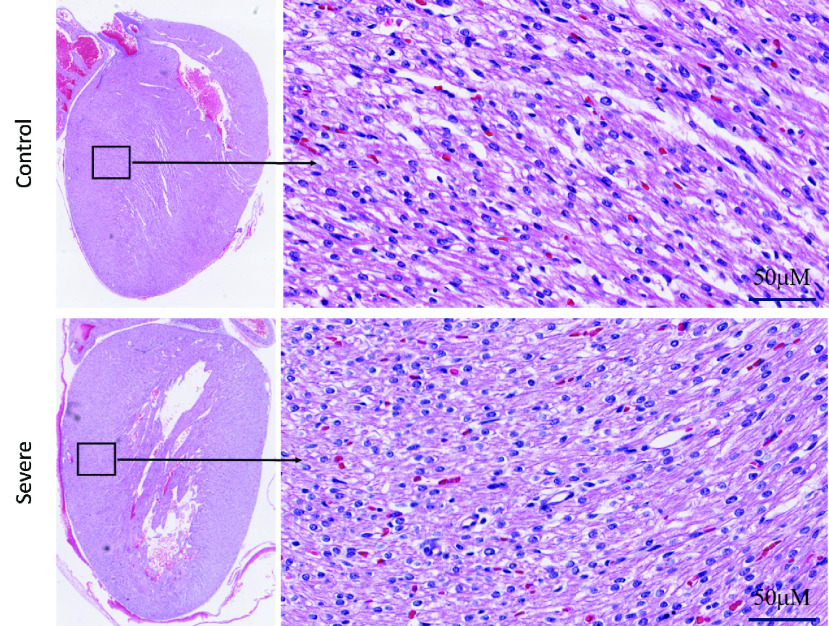
H&E evaluation of newborn heart tissue shows the healthy architecture and does not show any significant difference except a few apoptotic cells scattered in the severe (HADHA^e4−/−^) condition. The left panel is a $1.5\times $ magnification, and the right panel is $40\times $ magnification.

**FIGURE 5.**
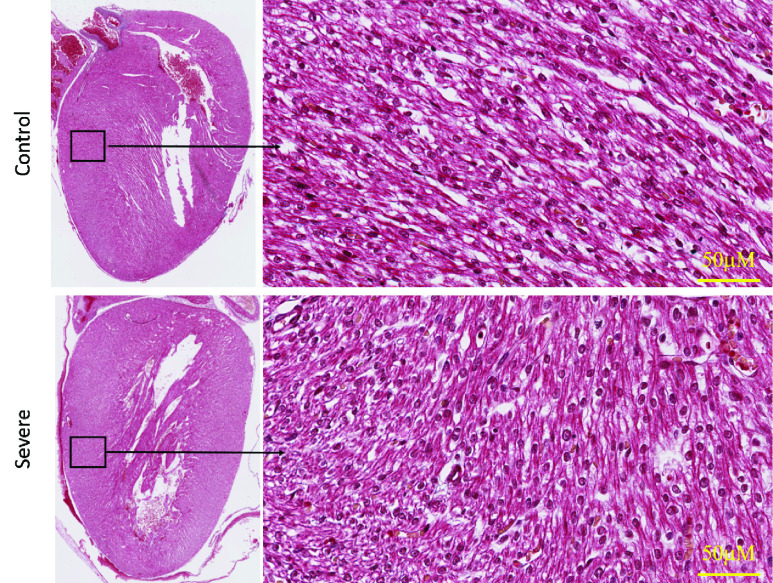
Trichrome evaluation of newborn heart tissue shows a healthy architecture and does not show any fibrosis indicating a structurally healthy heart. The left panel is a $1.5\times $ magnification, and the right panel is $40\times $ magnification.

Hematoxylin-Eosin of the newborn heart is illustrated in Figure 3. H&E evaluation of the heart tissue showed a few scattered apoptotic cells in severe (HADHA^e4−/−^) conditions; however, the overall tissue architecture reveals no significant difference between wild-type control and severe (HADHA^e4−/−^) heart. Moreover, in Figure 4, the trichrome staining of the heart with severe (HADHA^e4−/−^) condition shows no fibrosis, indicating a healthy organ structurally. We used translocase of outer mitochondrial membrane (TOMM40), a standard mitochondrial detection marker, to evaluate mitochondrial density in the heart tissue by immunohistochemistry (IHC). The quantification of antibody-mediated positive staining also shows insignificant differences in the organelle density in both wild-type and severe (HADHA^e4−/−^) conditions with a p-value of 0.19 (Figure 5).

**FIGURE 6.**
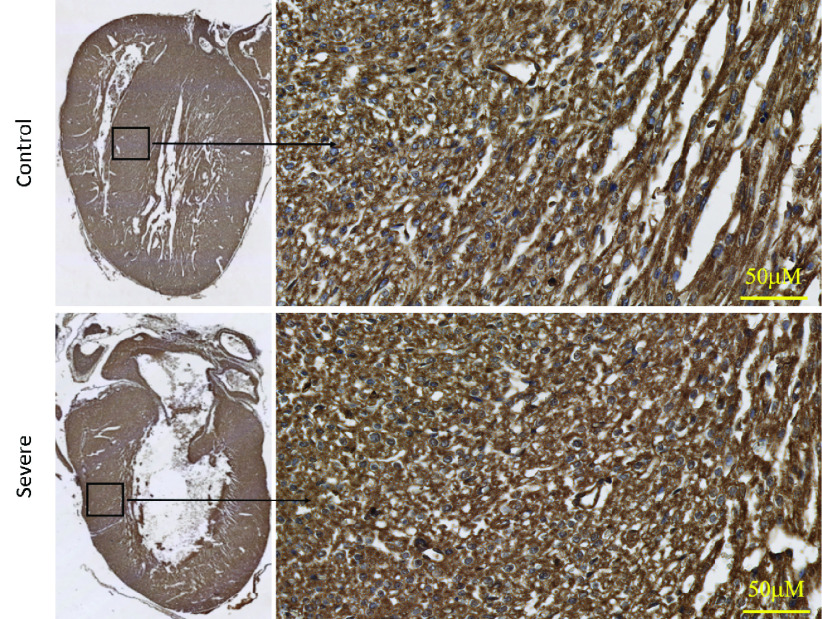
Heart tissue stained with TOMM40 using standard IHC method and DAB detection method. The left panel is a $1.5\times $ magnification, and the right panel is $40\times $ magnification. Mitochondrial staining and quantification show similar quantities of organelle in the newborn heart.

## IV. Discussion

This study provides the influencing role of a mutant trifunctional protein-encoding gene in cardiac and kidney mitochondrial redox state. Moreover, the result of the volumetric mitochondrial redox state, a bioenergetic parameter to address the mitochondrial health and function, is presented in three-dimension. We show that severe (HADHA^e4−/−^) conditions cause significant oxidation in heart and kidney tissue but at different levels. Since the heart depends heavily on mitochondrial integrity and function for its energy supply, extensive mitochondrial dysfunction could adversely affect heart function. We suggest that this extreme mitochondrial dysfunction could be a significant contributing factor to systemic heart failure and sudden death in severe (HADHA^e4−/−^) condition pups. Since we studied only the postnatal heart, which shows extensive oxidative status, but we do not know if the prenatal hearts are normal (future studies). However, it is worth noting that a reduction in the RR to 90–70% is sufficient to cause sudden death. Our wild-type control RR ranges between 0.943–1.427 while the severe (HADHA^e4−/−^) group is 0.75–0.92 suggesting anything below 0.92 could be detrimental to newborn survival.

FAO converts long-chain fatty acids into acetyl Co-A and acyl CoA in 4 steps by acyl Co-A dehydrogenase, enoyl Co-A hydratase, hydroxy acyl Co-A dehydrogenase, and ketoacyl Co-A thiolase. HADHA gene product is responsible for 3 of the four activities, and HADHB carries out thiolase activity. The catabolic process of $\beta $-oxidation is carried out by the hetero-octameric enzyme complex (4HADHA/4HADHB) with FADH_2_ produced in the first step by acyl-CoA dehydrogenase and NADH produced in the third step is hydroxy acyl CoA dehydrogenase step [1]. In a normal functioning cell, higher NADH/NAD+ levels inhibit FAO function in a feedback mechanism. The newborn heart relies heavily on aerobic metabolism for energy (ATP) supply which quickly switches to FAO post-natal. Since mitochondrial dysfunction has been reported in HADH subunit mutants, we used the novel cryoimaging / redox-ratio measurement method to evaluate mitochondrial functionality efficiency to understand the bioenergetic imbalances in our mutant animal model. For the first time, in this report, we show that mitochondrial dysfunction could be resulting from the disruption of FAO and ETC.

Using the 3D optical cryoimaging method to observe the mitochondrial redox state changes by recording the intensity of two autofluorescence signals originating from mitochondria (NADH and FAD) is a superior method to evaluate mitochondria. Snap freezing the tissue, imaging at ultra-low temperature, and measuring the autofluorescence ratio of endogenous components of the tissue ensures minimal sample manipulations gives us a reliable and accurate measure of the redox status of the tissue at the time of harvest. Along with the ability to minimize processing-induced variations, this method is also inclusive (data comes from structurally and functionally healthy and compromised organelles), thus giving the tissue’s exact overall redox status. Therefore, perturbations in NADH and FAD intensity reflect the level of mitochondrial dysfunction directly due to HADHA mutation. Large quantities of NADH and FADH_2_ are produced during each FAO cycle, and ETC uses these electron carriers to power oxidative phosphorylation, thus generating ATP [18], [32]. Therefore, any alternation in NADH and FAD pool could impair ATP production, which exacerbates mitochondrial dysfunction [21]. Thus, the observed decrease in the mitochondrial redox ratio reflects the consequence of HADHA mutation and the extent of impairment of ATP production that directly affects the organ function. It also underscores the complex interactions between mitochondrial coenzymes, energy production/supply, and organ failure.

Our 3D optical cryo-imaging results revealed significant oxidation in cardiac redox state due to more oxidized NADH and less reduced FAD. In other words, cardiac cells’ ability to maintain balanced NADH/FAD for normal bioenergetics is diminished after extreme mitochondrial dysfunction due to trifunctional protein mutation. The decrease in the NADH level can impair the TCA cycle, which supplies the reduced equivalents to produce ATP via OxPhos. Some of the byproducts of OxPhos activity are reactive oxygen species, and the elevation in ROS production may further affect the function of ETC, leading to accumulated damages to mitochondria [33]–[35]. The mutation changed the RR remarkably in kidneys but not to the extent we observe in the heart tissue. These changes can be associated with mitochondria dysfunction, impaired ETC function, and more than the usual amount of free radical production. In kidneys, the ATP generation source may be more dispensable and have a cell-type-dependent mechanism. In other words, ATP can produce through aerobic respiration in cells that have a higher demand for oxygen, including proximal tubules in the renal cortex, or through both aerobic and anaerobic respiration in cells that require lower O_2_ supply, such as glomerular cells [10], [36]. Our results show a decrease in the cortical region’s RR while the medullary redox ratio is relatively high. The severe (HADHA^e4−/−^) condition group’s cortical region reflects impaired ATP production because of decreased oxidation of the NADH and FADH_2_ and defective OxPhos. In contrast, the high RR in the medullary region could be attributed to normal ATP production because of anaerobic respiration.

**FIGURE 7.**
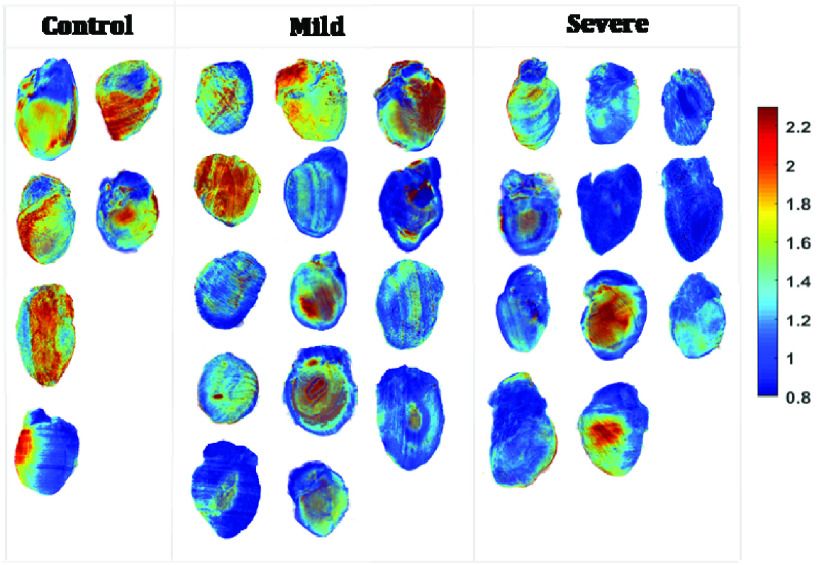
3D optical cryoimages of the mitochondrial redox ratio in the individual hearts (n = 6/control, n = 14/mild (HADHA^e4+/−^) and n = 11/severe (HADHA^e4−/−^)) for both groups. Statistical analysis shows a significant difference between control vs. severe (HADHAe4−/−), and control vs. mild (HADHAe4+/−) with p-value of 5.56e-7 and 0.031 using one-way ANOVA followed by post hoc, respectively.

**FIGURE 8.**
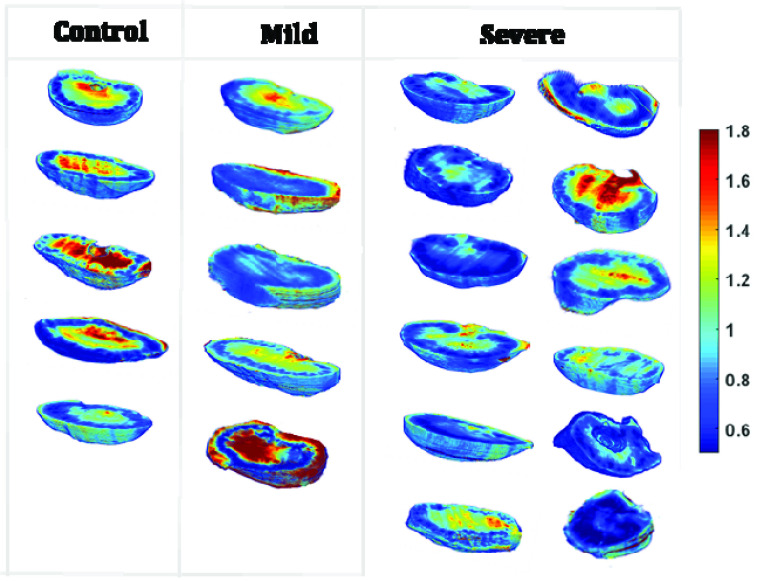
3D optical cryoimages of mitochondrial redox ratio in individual kidneys (control, n = 5, mild (HADHAe^4+/−^), n = 5 and severe (HADHA^e4−/−^), n = 12). One-way ANOVA followed by post hoc showed significant difference between control vs. severe (HADHAe4−/−) with p-value of 0.023 and no significant difference was detected between control and mild (HADHAe4+/−) (p = 0.268).

Our data suggest that the severe (HADHA^e4−/−^) mutants having the reduced mitochondrial function (representing both ETC and FAO activities) could be a contributing factor for neonatal mortality while the healthy surviving littermate heterozygotes and wild-type controls having fully functional mitochondria. Interestingly since the estimation of mitochondrial content by Tomm40 staining was statistically identical, which means that most of these mitochondria in the homozygous mutants are either defective or nonfunctional. Further, redox ratio estimations in the mitochondrially dense (second only to the heart) kidney seem to show less significant difference among the various genotypes; Wild-type controls and mild (HADHA^e4+/−^) condition group compared to severe (HADHA^e4−/−^) group (Figure 7 and 8), suggesting cardiac mitochondrial dysfunction could precede kidney mitochondrial dysfunction. The heart and kidneys were isolated at birth (3–6hrs), and since cardiac dysfunction, as measured by redox ratio, was apparent at this time point, and it coincides with neonatal deaths in the severe (HADHA^e4−/−^) group (lower RR levels), it strongly suggests that heart failure due to dysfunctional heart mitochondria is the reason for HADHA mutants neonatal death. Our redox-ratio evaluation does not pinpoint a specific pathway (FAO, ETC, or both) that is severely affected as it measures the overall NADH/FAD levels but certainly differentiates which organ is most affected among the mitochondrial rich heart and kidney. Previous reports have suggested that during cardiac arrest and resuscitation, cardiac mitochondria functions differently from kidney, liver, and brain mitochondria [37]. Our study demonstrated that HADHA mutation diminishes mitochondrial function and contributes potentially to the organ (heart) failure and sudden death. Mitochondrial isolation and assay methods tend to enrich healthy mitochondria and possibly could underestimate (skewed data) the extent of dysfunction, and thus our in-situ redox measurements give an accurate, unaltered picture of the organ’s mitochondrial function.

## V. Conclusion

The current study investigated the contribution of HADHA encoded trifunctional protein mutation in cardiac and renal mitochondrial metabolism. We observed a significant difference in heart and kidney redox state of mitochondrial between severe (HADHA^e4−/−^) transgenic mutant and wild-type rats with heart tissue displaying the most significant difference. Our 3D redox measurement data gives an accurate, unaltered view of the organ’s mitochondrial function.

## Appendix

The redox ratio images of all hearts and kidneys samples of control, mild (HADHA^e4+/−^) and severe (HADHA^e4−/−^) groups are shown in Figure 7 and Figure 8 as reference.
